# Supplementary material for: MetaRibo-Seq measures translation in microbiomes
Source: Nat Commun. 2020 Jun 29;11:3268. doi: 10.1038/s41467-020-17081-z (PMC7324362; doi:10.1038/s41467-020-17081-z)
Supplement: Supplementary file 10 — Supplementary Data 7 [file 41467_2020_17081_MOESM10_ESM.zip › File2/Confidence_High_Taxonomy/57750_out.krona.html]

Javascript must be enabled to view this page.

members
magnitude
magnitudeUnassigned
count
unassigned
taxon
rank

57750\_out

6

superkingdom
6
2

phylum
1239
6

186801
1
class

order
1
186802

species
1
1898207

SRS024132\_contig\_number\_42115

species

SRS011239\_contig\_number\_27754SRS013476\_contig\_number\_38873SRS016203\_contig\_number\_626SRS078419\_contig\_number\_31983SRS104400\_contig\_number\_43084
5
1263026
